# The influence of family socio-economic status on learning engagement of college students majoring in preschool education: The mediating role of parental autonomy support and the moderating effect of psychological capital

**DOI:** 10.3389/fpsyg.2022.1081608

**Published:** 2023-01-09

**Authors:** Yuan Qiu, Pingzhi Ye

**Affiliations:** School of Education, Guangzhou University, Guangzhou, China

**Keywords:** college students majoring in preschool education, family socio-economic status, learning engagement, parental autonomy support, psychological capital

## Abstract

**Objective:**

To explore the relationship between family socio-economic status and learning engagement of college students majoring in preschool education, as well as the mediating role of parental autonomy support and the moderating role of psychological capital.

**Methods:**

A sample of 986 college students majoring in preschool education from Guangdong Province and Jiangxi Province were investigated by family socio-economic status questionnaire, learning engagement questionnaire, parental autonomy support questionnaire and psychological capital questionnaire.

**Results:**

First, there was a significant positive correlation between family socio-economic status and learning engagement (*β* = 0.09, *p* < 0.05). Second, parental autonomy support played a completely mediating role between family socio-economic status and learning engagement (*β* = 0.05, *p* < 0.01). Third, the relationship between parental autonomy support and learning engagement was moderated by psychological capital, and students with high psychological capital had higher learning engagement (*β* = 0.24, *p* < 0.001).

**Conclusion:**

Family socio-economic status could directly affect learning engagement, and could also indirectly affect learning engagement through psychological capital level moderating parental autonomy support. Our findings highlight the importance of creating an autonomous supportive family environment and focusing on the learning of students from low socio-economic status. Meanwhile, stimulating students’ psychological capital should be regarded as a part of education and teaching.

## 1. Introduction

Learning engagement was an important influencing factor in students’ learning process, and it has increasingly become a prominent issue in the field of college students’ learning and development. Schaufeli et al. (2002) first put forward the concept of “Learning Engagement” and defined it as the emotional state that learners keep positive, energetic and focused in the learning process; its main features are energy, dedication and concentration.

The research shows that learning engagement has a positive predictive effect on college students’ personal cognitive development, learning satisfaction, critical thinking and academic achievements, etc. (Xie et al., 2020). It was found that there was a positive correlation between learning engagement and academic achievement, that is, when learners devote more learning time, their academic achievements will be higher (Salanova et al., 2010). Learning engagement, as an important predictor of academic achievement, could not only provide a reference for judging the quality of individual learning, but also affect individual growth and future development (Christenson et al., 2012). However, the influence of external factors on learning engagement is still an unresolved issue. In addition to school, how family environment affects college students’ learning engagement remains to be further explored. Preschool education is an important part of the national education system and the beginning of life-long education. College students majoring in preschool education are important reserve talents for future preschool teachers. Therefore, this study takes college students majoring in preschool education as the participants to explore the influence of family socio-economic status (family SES) on learning engagement.

### 1.1. Family SES and learning engagement

Family SES was of great significance to the growth and development of individuals. It refers to the ranking according to the value resources (such as education, wealth) owned by families (generally divided into high, medium and low levels). It represents a comprehensive indicator of a stable and observable family’s physical environment (Matthews and Gallo, 2011), parents’ occupation education level and income are its main indicators (Zhong and Huang, 2012). Ecological Systems Theory (EST) holds that the family was an important micro-system affecting the development of students, which was not only the main social background of students’ growth and development, but also the first place of their psychological development (Zhou et al., 2018). The family SES as an exosystem other than microsystem and mesosystem, may have an impact on students’ learning engagement level.

Existing research indicates that family SES could positively predict students’ level of learning engagement (Shi et al., 2013), which has a great impact on students’ academic achievement and the development of various abilities to obtain academic achievement (Brito and Noble, 2014). The Positive Development Theory has always emphasized that family SES plays an important role in the growth of teenager (Atkiss et al., 2011). The influence of family SES on teenagers was more reflected in the degree of parents’ participation in teenagers’ learning or life. Lee and Bowen (2006) studied different levels of family SES, and found that the higher the family SES, the higher the parents’ expectations of their children. Meanwhile, the closer the relative parent–child relationship is, the closer the cooperation between home and school will be. It shows that parents with high family SES are more willing to spend more time in their children’s learning activities and urge their children to study harder. Relevant research has found that students with different family SES have different family resources and opportunities, which will also have an impact on students’ growth and development (Tucker-Drob and Harden, 2012). Compared with families with lower family SES, parents with higher family SES could provide more valuable resources for their children and promote their learning engagement through material and psychological investment (Bi et al., 2018). In addition, some scholars (Pang et al., 2013) have also found through the study of family SES that the education level of parents and the learning aids provided by parents for children are the most influential factors on students’ learning ability. Meanwhile the degree of correlation between family wealth and learning achievement depends on the parents’ support for children’s learning. It could be inferred that different family SES will have different effects on students’ learning engagement. Therefore, this study proposes hypothesis 1 (H_1_): Family SES would positively predict students’ learning engagement.

### 1.2. The mediating role of parental autonomy support

Parental autonomy support was also an important factor affecting students’ learning engagement. Parental autonomy support is a kind of parenting style that regards individuals as a social environment with self-determination (Moreau and Mageau, 2012). It means that parents can accept their children’s emotions, opinions and reactions, provide them with relevant information and support their independent choice and self-determination, and help them explore and practice their personal values and interests (Wang et al., 2007). The Self-determination Theory (SDT) proposed by American psychologists Deci and Ryan in the 1980s emphasized that autonomy support was one of the important social environmental factors that affect learners’ learning. They define it as an individual’s ability to feel the support of parents, family members, relatives and friends and other significant others when making independent decisions, and collect more valuable information (Deci and Ryan, 1987).

According to SDT, a supportive external environment could meet the basic psychological needs of individuals, while non supportive environments such as compulsion and control were the opposite (Vansteenkiste and Ryan, 2013). According to available research (Soenens and Vansteenkiste, 2010) the satisfaction of the three basic needs, namely, individual autonomy needs, ability needs and relationship needs, is inseparable from the supportive environment. It was beneficial to stimulate the individual’s intrinsic motivation, while the non-supportive environment not only inhibits individual’s intrinsic motivation, but also causes individual’s psychological and behavior problems. According to the EST (Zhou et al., 2018), the growth and development of individuals are affected by the interaction of their internal factors and external environment. Deci and Ryan (2012) pointed out that supportive environment mainly refers to the effective interaction between individuals and important others in the external environment (such as parents, teachers) through establishing close interpersonal relationships. It has been found that learning engagement was just the “mechanism” of the interaction between individual internal factors and external environment (Schaufeli et al., 2002). As important others of students, parental autonomy support helps to stimulate students ‘internal motivation and promote learning engagement. Some studies have shown that students who grow up in the environment of parents’ autonomous support are more active in learning than students who grow up in the environment of nonparents’ autonomous support (Grolnick, 2009). Gillet et al. (2011) also agreed with this view and proposed that students growing up in this environment have stronger learning opportunities and could achieve higher academic performance. Through research, Vasquez et al. (2016) further found that parental autonomous support not only benefits children’s motivation development, but also keeps them in a positive psychological state. The environment of parental autonomy support could provide children with a harmonious, friendly and democratic growth atmosphere. Students growing up in this environment could feel the positive energy of their parents’ trust, encouragement and support, so as to enhance their internal motivation and make them willing to invest more time and energy in the learning process.

In addition, the Family Stress Model holds that parents with low family SES (such as financial difficulties) were more likely to have bad emotions or behavior problems due to unfavorable situations, thus lowering the quality of parenting (for example, reduced family warmth and increased family conflict; Conger and Donnellan, 2007; Masarik and Conger, 2017). This leads parents to adopt controlled parenting styles more often and lack support for teenagers’ independent needs, which will eventually affect their positive development. Empirical studies have also found that parents with lower family SES pay less attention to their children (Bae and Wickrama, 2014), have more negative parenting styles, and give their children less warmth, understanding and autonomous needs (Zhang et al., 2017). It was concluded that there may be a close relationship between family SES and parental autonomy support. Considering the existing studies on the relationship between family SES and parental autonomy support, as well as the relationship between autonomy support and students’ learning engagement, this study proposes hypothesis 2 (H_2_): Parental autonomy support would play a mediating role in the relationship between family SES and learning engagement.

### 1.3. The moderating role of students’ psychological capital

With the rapid development of positive psychology, more and more researches have begun to pay attention to the influence of positive factors on individual behavior. Although family SES may affect students’ learning engagement through parental autonomy support, there may be individual factors in this process. By combing the existing research literature, this study holds that psychological capital plays a moderate role in this process (Xie et al., 2022). Psychological capital refers to a positive psychological state that individuals reflect in the process of self-growth and development, which is an inherent positive resource with the effect of supplementing energy and stimulating motivation (Luthans et al., 2006).

On the one hand, according to the Conservation of Resources Theory (COR), psychological capital, as a positive psychological quality of individuals, could provide resources and supplement energy for their energy consumption process (Schaufeli and Bakker, 2004), and has a positive effect on individual behavior. Specifically, in the process of college study, students will face various learning difficulties and tasks, and will also show various academic emotions. Individuals with high psychological level were better at coping with negative academic emotions (Wang et al., 2021) and academic pressure (Konrad et al., 2022), thus showing a higher level of learning engagement (Lin, 2020). On the contrary, individuals with low psychological capital levels were more likely to show procrastination (Saman and Wirawan, 2021) and academic burnout (Zhang et al., 2021). It can be seen that psychological capital may be the protective factor of learning engagement.

On the other hand, according to Developmental Situation Theory, the interaction between individuals and their environment could affect their behavior (Lerner, 2006). That is, psychological capital (individual factors) and parental autonomy support (environmental factors) work together on individual behavior (learning engagement). Psychological capital and parental autonomy support were positive resources for individual learning engagement, which could have a positive effect on individual learning engagement. Specifically, when the level of parental autonomy support was high, individuals with higher psychological capital had stronger internal learning motivation, and were more willing and active in learning activities. In other words, individuals with high psychological capital may have a higher level of learning engagement than individuals with low psychological capital. It could be inferred that psychological capital, as a positive psychological resource, has an important influence on the relationship between parental autonomy support and learning engagement. Therefore, this study puts forward hypothesis 3 (H_3_): Psychological capital may moderate the relationship between parental autonomy support and learning engagement.

### 1.4. The present study

The purpose of this study was to explore the relationship and influence between family SES and learning engagement of college students majoring in preschool education. Through combing the existing research results, it was found that parental autonomy support and psychological capital were also important factors that affected college students’ learning engagement. However, there was no research to show whether parental autonomy support and psychological capital work at the same time in the special group of college students majoring in preschool education. If so, what are their respective roles? Mediating or moderating role? All these problems need to be further discussed and solved in this research. Based on the existing research results, this study puts forward the following three hypotheses.

*Hypothesis 1* (H_1_): Family SES would positively predict students’ learning engagement.

*Hypothesis 2* (H_2_): Parental autonomy support would play a mediating role in the relationship between family SES and learning engagement.

*Hypothesis 3* (H_3_): Psychological capital may moderate the relationship between parental autonomy support and learning engagement.

**Figure 1:**
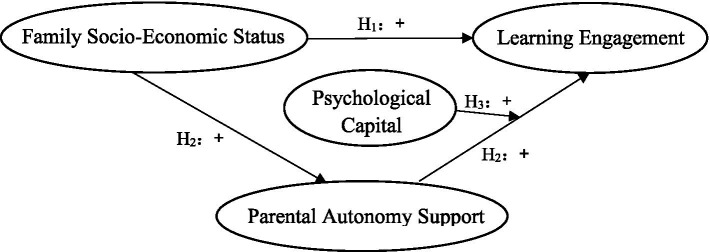
Family SES, learning engagement, parental autonomy support and psychological capital relationship hypothesis model diagram. H_1-3_ represents hypothesis 1–3 respectively, and the symbol represents the direction of influence.

Based on the hypothesis put forward in this paper, this study constructed a moderated mediating model (see Figure 1) to explore the relationship between family SES and learning engagement, as well as the mediating role of parental autonomy support and the moderating role of psychological capital.

## 2. Materials and methods

### 2.1. Participants

Two public normal universities in Guangdong Province and one public normal university in Jiangxi Province with pre-school education specialty were selected as the survey schools. The method of cluster random sampling was adopted to distribute paper questionnaires by class. A total of 1,072 questionnaires were distributed, 1,050 of which were recovered, with a recovery rate of 97.95%. After removing 64 invalid questionnaires, 986 were valid, with an effective rate of 93.90%. Relevant statistical information of the sample, including gender, age, whether the only child, grade, family location, etc. (See Table 1) Among them, the average age of the participants was 19.76 ± 1.29 years old. All procedures followed were in accordance with the ethical standards of the responsible Committee on Human Experimentation [Guangzhou University, Guangdong Province, China] and with the Helsinki Declaration of 1975, as revised in 2000. Written informed consent to participate in this study was provided by the participants.

**Table 1:** Demographics of participants (*N* = 986).

**Variable**	**Group**	***N***	**%**
Gender	Male	52	5.27
	Female	934	94.73
Age	18 and Below	170	17.24
	19–20	544	55.17
	21–22	252	25.56
	23 and Above	20	2.03
Only child or not	Yes	164	16.63
	No	822	83.37
Grade	Freshmen	350	35.50
	Sophomores	301	30.53
	Junior	243	24.65
	Seniors	92	9.33
Home location	City	236	23.94
	Countryside	750	76.07
Class cadre or not	Yes	353	35.80
	No	633	64.20
Voluntary choice before entering school	Independent choice	540	54.77
	Willingness of parents and others	175	17.75
	Dispense	271	27.49
Degree of understanding of the major before admission	Do not understand	356	36.11
	Have some understanding	617	62.58
	Very understanding	13	1.32
Father’s educational background	Junior high school and below	620	62.88
	High school / technical secondary school	255	25.86
	Junior college students	52	5.27
	Undergraduate	56	5.68
	Postgraduate (Master or Phd)	3	0.30
Mother’s educational background	Junior high school and below	728	73.83
	High school / technical secondary school	179	18.15
	Junior college students	40	4.06
	Undergraduate	38	3.85
	Postgraduate (Master or Phd)	1	0.10
Father’s occupation	Workers, farmers, unemployed (including housewives), etc.	528	53.55
	Self-employed, service personnel (e.g., waiters, drivers, etc.)	241	24.44
	Enterprise staff, junior professional technicians, small business owners, etc.	72	7.30
	Intermediate manager / technician, party and government / public institution general staff	84	8.52
	Middle and senior leadership of party and government / public institution, senior academic expert / administrator / technician, private entrepreneurs, etc.	20	2.03
	Others	41	4.16
Mother’s occupation	Workers, farmers, unemployed (including housewives), etc.	623	63.19
	Self-employed, service personnel (e.g., waiters, drivers, etc.)	207	20.99
	Enterprise staff, junior professional technicians, small business owners, etc.	53	5.38
	Intermediate manager / technician, party and government / public institution general staff	62	6.29
	Middle and senior leadership of party and government / public institution, senior academic expert / administrator / technician, private entrepreneurs, etc.	10	1.01
	Others	31	3.14
Total annual household income	50,000 and below	423	42.90
	50,000–100,000	337	34.18
	100,000–150,000	140	14.20
	150,000–200,000	49	4.97
	200,000 and above	37	3.75

### 2.2. Measures

#### 2.2.1. Family SES questionnaire

The measurement indicators of family SES include parents’ occupation, parents’ educational level and total annual family income (Liu et al., 2020). Considering the actual situation, parental occupation status was coded into five categories (1–5) from “workers, farmers, unemployed (including housewives)” to “senior managers and senior professionals.” Parents’ educational background levels were scored on 1–5 scale ranging from “junior high school and below” to “graduate degree and above.” Annual family income after tax was measured on five levels (1–5) from “less than Chinese ¥50,000″ (approximately US $6,982) to “more than Chinese ¥ 200,000″ (approximately US $27,931). According to the PISA index of ESCS [Economic, Social and Cultural Status; Organisation for Economic Co-operation and Development (OECD), 2014], principal component analysis was performed on standardized variables (average scores of parents’ education level, parents’ occupation, and family annual income) to calculate the total score of family SES. The higher the average score, the higher the family SES level. Because the range of parents’ occupation was not ideal, only parents’ educational level and annual family income were analyzed. During data processing, parents’ educational background and annual family income were transformed into standardized statistical analysis, which was used as the measurement index of family SES. The total Cronbach’s alpha of the questionnaire was 0.70.

#### 2.2.2. Learning engagement questionnaire

The college students’ learning engagement questionnaire compiled by Liao (2011) was adopted. The questionnaire consists of 20 items in three dimensions: Behavioral engagement, cognitive engagement and emotional engagement. The questionnaire adopts Likert’s five point scoring method, from one to five, it means “completely inconsistent” to “completely consistent.” The higher the average score, the higher the degree of learning engagement. The total Cronbach’s alpha of the questionnaire was 0.95, and the three dimensions of Cronbach’s alpha were 0.84, 0.89, and 0.87, respectively.

#### 2.2.3. Parental autonomy support questionnaire

In this study, the Parental Autonomy Support Scale revised by Wang et al. (2007) was adopted, with 12 items in total. The questionnaire was scored on Likert’s five-point scale, ranging from one to five, indicating “completely inconsistent” to “completely consistent.” The higher the average score, the higher the degree of parental autonomy support that children could perceive. The total Cronbach’s alpha of the questionnaire was 0.92.

#### 2.2.4. Psychological capital questionnaire

The Psychological Capital Questionnaire used in this study was mainly based on the Psychological Capital Questionnaire (PCQ-24) prepared by foreign scholars Luthans et al. (2007) and the adolescent psychological capital questionnaire prepared by domestic scholars Ye and Fang (2015). The questionnaire consists of 22 items in four dimensions of hope, optimism, self-confidence and resilience. The questionnaire adopts Likert’s five point scoring method (from one to five, it means “completely inconsistent” to “completely consistent”). The higher the average score, the higher the level of psychological capital. The total Cronbach’s alpha of the questionnaire was 0.92, and the Cronbach’s alpha of the four dimensions were: 0.92, 0.87, 0.78, 0.79.

### 2.3. Procedure

Firstly, Harman single factor test was used to test the bias of common methods in variable items. Secondly, the correlation between family SES, parental autonomy support, learning engagement and psychological capital was investigated. After verifying the significant correlation and regression between the four variables, the structural equation model of maximum likelihood estimation was used to verify the mediating and moderating effects between family SES and learning engagement.

SPSS (Version 25.0) and Amos (Version 24.0) were used for data analysis.

## 3. Results

### 3.1. Testing of common method bias

In this study, anonymous questionnaires were filled in, and some items were reverse expressed. Control the common method biases that may exist in the program. The method of Harman single factor test was used to make an exploratory factor analysis of the items of variables without rotation. The results showed that the eigenvalues of nine factors were greater than one, and the variance explained by the first factor was 32.25%, less than the critical value of 40% (Zhou and Long, 2004). Therefore, there was no obvious common method bias in this study.

### 3.2. Description statistics and correlation matrix

Descriptive statistics and correlation analysis results were shown in Table 2. Correlation analysis showed that only child or not, grade, family location, the voluntary choice before entering school, and the degree of understanding of the major before admission were significantly correlated with parental autonomy support. Grade, class cadre or not, and the degree of understanding of the major before admission were significantly related to psychological capital. Class cadre or not and the degree of understanding of the major before admission were significantly concerned with the learning engagement. Age, only child or not, home location and the degree of understanding of the major before admission were significantly correlated with the family SES.

**Table 2:** Mean, standard deviation and correlation analysis of variables (*N* = 986).

**Variable**	***M***	***SD***	***K***	***W***	**1**	**2**	**3**	**4**	**5**	**6**	**7**	**8**	**9**	**10**	**11**	**12**
**Gender**	1.95	0.22	14.10	−4.01	1											
**Age**	19.76	1.29	0.06	0.44	−0.04	1										
**Grade**	2.08	0.99	−0.94	0.43	−0.04	0.78^**^	1									
**Home location**	2.28	0.83	−1.30	−0.57	0.09^**^	0.09^**^	−0.03	1								
**Only child or not**	1.83	0.37	1.22	−1.80	0.09^**^	0.08^*^	−0.04	0.37^**^	1							
**Class cadre or not**	1.64	0.48	−1.65	−0.59	0.02	−0.05	−0.10^**^	0.09^**^	0.03	1						
**Voluntary choice before entering school**	1.73	0.87	−1.44	0.56	−0.10^**^	0.09^**^	0.10^**^	−0.01	0.06	0.01	1					
**Degree of understanding of the major before admission**	1.65	0.50	−1.16	−0.33	0.11^**^	−0.12^**^	−0.13^**^	−0.07^*^	−0.13^**^	0.02	−0.27^**^	1				
**Family SES**	1.62	0.70	2.25	1.53	−0.05	−0.13^**^	0.03	−0.50^**^	−0.42^**^	−0.05	−0.03	0.11^**^	1			
**Learning engagement**	3.04	0.65	0.26	0.41	−0.03	0.04	0.03	−0.01	−0.05	−0.07^*^	−0.01	0.15^**^	0.08^*^	1		
**Psychological capital**	3.41	0.55	0.20	0.20	−0.04	−0.03	−0.08^*^	−0.06	−0.06	−0.08^*^	−0.03	0.18^**^	0.09^**^	0.70^**^	1	
**Parental autonomy support**	3.53	0.73	0.01	−0.28	−0.01	−0.05	−0.07^*^	−0.06^*^	−0.11^**^	−0.01	−0.10^**^	0.09^**^	0.12^**^	0.46^**^	0.47^**^	1

^*^p < 0.05, ^**^p < 0.01, ^***^p < 0.001. *K* represents Kurtosis, *W* represents Skewness. Numbers 1–12 represent different variables as shown below. 1 represents gender. 2 represents age. 3 represents grade. 4 represents home location. 5 represents only child or not. 6 represents class cadre or not. 7 represents voluntary choice before entering school. 8 represents degree of understanding of the major before admission. 9 represents family SES. 10 represents learning engagement. 11 represents psychological capital. 12 represents parental autonomy support.

Family SES was positively correlated with learning engagement, psychological capital and parental autonomy support. Learning engagement was positively correlated with psychological capital and parental autonomy support. There was a significant positive correlation between psychological capital and parental autonomy support.

### 3.3. Relationship between family SES and learning engagement

Without considering parental autonomy support, this study first constructs an impact model of the relationship between family SES and learning engagement. Structural equation model analysis: *X^2^/df* = 3.28, GFI = 0.99, IFI = 0.99, TLI = 0.99, CFI = 0.99, RMSEA = 0.05, in line with psychometric standards, and the model fitting was good. The direct effect of family SES on learning engagement was significant(*β =* 0.09, *p* < 0.05), hypothesis 1 was verified.

### 3.4. Effect of parental autonomy support on family SES and learning engagement

**Figure 2:**
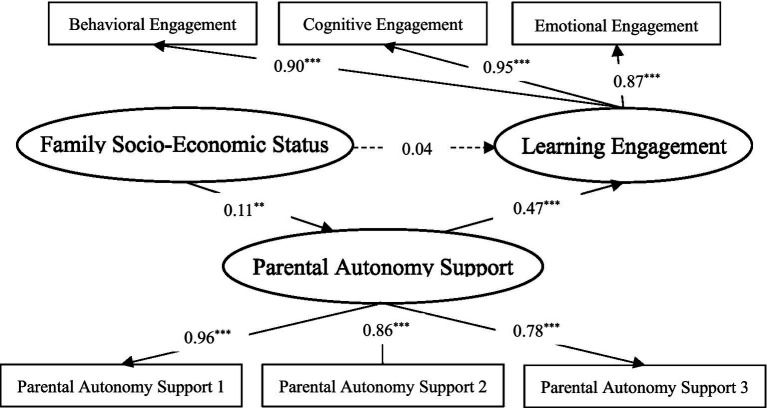
Standardized model diagram of the mediating role of parental autonomy support between family SES and learning engagement. ^*^p < 0.05, **p < 0.01, ^***^p < 0.001.

In order to further investigate the mediating role of parental autonomy support between family SES and learning engagement, the bootstrap program in Amos24.0 statistical software (Mackinnon, 2008) was used to extract 5,000 perform bootstrap samples from the original data (*N* = 986) by repeated random sampling. The 95% confidence interval of mediating effect estimated by bias-corrected was constructed. If the interval does not include zero, the mediating effect was significant. As shown in Figure 2. Structural equation model analysis: *X^2^/df* = 3.88, GFI = 0.98, IFI = 0.99, TLI = 0.98, CFI = 0.99, RMSEA = 0.05, in line with psychometric standards, the model fits well.

In the model, the path coefficient between family SES and parental autonomy support (*β* = 0.11, *p* < 0.01), parental autonomy support and learning engagement (*β* = 0.47, *p* < 0.001) was significant. After adding parental autonomy support, the direct action path between family SES and learning engagement changes from significant to insignificant (*β* = 0.26, *p* > 0.05, 95%CI[−0.03, 0.11], including zero). The mediating effect of parental autonomy support between family SES and learning engagement was significant (the effect value was 0.05, *p* < 0.01, 95%CI[0.02, 0.08], excluding zero), which indicates that parental autonomy support plays a complete mediating role between family SES and learning engagement. Hypothesis 2 was verified.

### 3.5. Relationship between family SES, parental autonomy support, learning engagement and psychological capital

**Figure 3:**
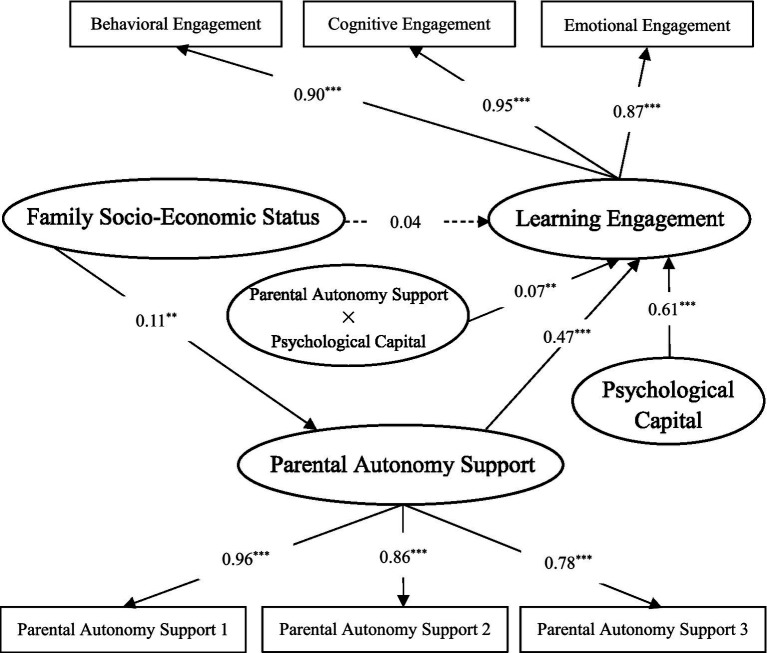
Standardized model of psychological capital moderating the relationship between parental autonomy support and learning engagement. ^*^p < 0.05, ^**^p < 0.01, ^***^p < 0.001.

In this study, family SES was used as the independent variable, learning engagement as the dependent variable, parental autonomy support as the mediator variable, and psychological capital as the moderating variable to test the moderated mediation model. According to Wen and Ye (2014), the parameters of the three regression equations need to be estimated for the mediated model with moderation. Before the estimation of each equation, all variables were standardized, and the variable variance inflation factor of all variables was not higher than three, so there was no serious multicollinearity problem. The results showed that (see Table 3) the interaction terms between family SES and psychological capital cannot significantly predict learning engagement (*β* = −0.04, *p* > 0.05) and parental autonomy support (*β* = −0.02, *p* > 0.05). The interaction between parental autonomy support and psychological capital has a significant impact on learning engagement (*β* = 0.07, *p* < 0.01). This indicates that psychological capital has a significant positive moderating effect on the relationship between parental autonomy support and learning engagement (see Figure 3). To sum up, parental autonomy support mediates the relationship between family SES and learning engagement, and psychological capital could moderate the second half of the path. Hypothesis 3 was verified.

**Table 3:** A test on the moderated mediating effect of family SES on learning engagement.

	**Equation 1 (Criterion: learning engagement)**		**Equation 2 (Criterion: parental autonomy support)**		**Equation 3 (Criterion: learning engagement)**	
	***SE***	***β***	***SE***	***β***	***SE***	***β***
Age	0.04	−0.01	0.05	0.02	0.04	−0.01
Grade	0.04	0.10^**^	0.05	−0.05	0.04	0.10^**^
Home location	0.03	0.06^*^	0.03	0.01	0.03	0.06^*^
Only child or not	0.03	−0.01	0.03	−0.07^*^	0.03	0.00
Class cadre or not	0.02	−0.01	0.03	0.03	0.02	−0.02
Voluntary choice before entering school	0.02	0.02	0.03	−0.09^**^	0.02	0.03
Degree of understanding of the major before admission	0.02	0.04	0.03	−0.04	0.02	0.05
Family SES	0.03	0.04	0.04	0.06	0.03	0.03
Psychological capital	0.02	0.70^***^	0.03	0.46^***^	0.03	0.61^***^
Family SES × psychological capital	0.02	−0.04	0.03	−0.02	0.02	−0.04
Parental autonomy support					0.03	0.19^***^
Parental autonomy support × psychological capital					0.02	0.07^**^
*R* ^2^	0.53		0.24		0.53	
*F*	91.62^***^		30.02^***^		91.62^***^	

^*^p < 0.05, ^**^p < 0.01, ^***^p < 0.001.

**Figure 4:**
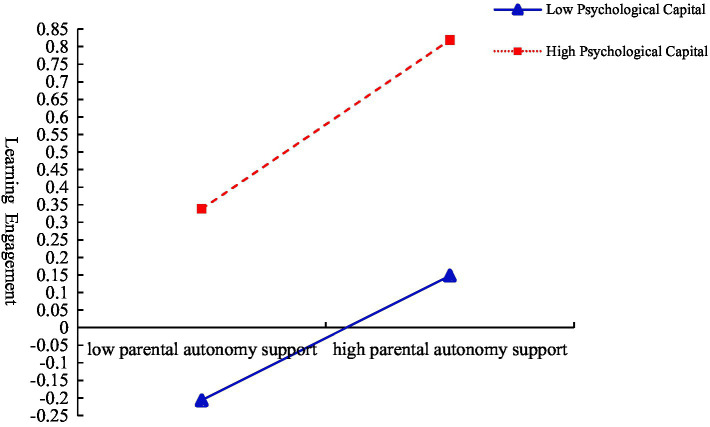
Moderation of psychological capital on the relationship between parental autonomy support and learning engagement.

In order to more clearly reveal the moderating trend of psychological capital between parental autonomy support and learning engagement, the method of Dearing and Hamilton (2006) was used. According to the scores of learning engagement corresponding to the positive and negative standard deviation of parental autonomy support in different psychological capitals, the interactive effect diagram was drawn (see Figure 4). Simple slope test shows that when the level of psychological capital is high, parental autonomy support has a relatively strong effect on promoting learning engagement (*B_simple_* = 0.24, *SE* = 0.04, *p* < 0.001). On the contrary, when the level of psychological capital is low, parents’ autonomy support has a relatively weak effect on promoting learning engagement (*B_simple_* = 0.11, *SE* = 0.03, *p* < 0.001).

## 4. Discussion

This study explored the relationship and influence between family SES and learning engagement through 986 participants. The results showed that: (a) Family SES significantly positively predicted learning engagement. (b) Parental autonomy support plays a mediating role between family SES and learning engagement. (c) Psychological capital moderates the relationship between parental autonomy support and learning engagement.

### 4.1. Relationship between family SES and learning engagement

The direct effect verifies the hypothesis 1 of this study. Family SES positively predicts the level of learning engagement. According to EST (Zhou et al., 2018), family was an important microsystem affecting students’ development, and family SES, as an important background index of students’ original family, will have an important impact on their learning engagement.

Students who grow up in families with higher Family SES will feel higher expectations from their parents, and their parents will also pay more attention to them (Lee and Bowen, 2006). Generally speaking, the living standard in cities is higher than that of the countryside. It also means that parents of students in cities could give them better living conditions and educational resources. On the contrary, rural families are relatively weak in supporting their children in various aspects. From the perspective of family education investment, families with high family SES are more willing to invest more resources in all aspects of their children’s education (Lareau, 2011). Gollnick and Chinn (2017) showed that families with higher family SES could provide high-quality resources for children’s education in all aspects, making their children have an advantage in education. Family Investment Theory (Matthews and Gallo, 2011) also confirmed that families with high family SES were willing to provide more capital support for their children’s educational development, which will have an important impact on their children’s learning, life and other aspects. Therefore, compared with students from low family SES families, students from high family SES families could avoid a lot of pressure from life and have more time and energy to study.

### 4.2. Effect of parental autonomy support on family SES and learning engagement

The mediating effect verified hypothesis 2 that parental autonomy support plays a mediating role in the relationship between family SES and learning engagement. Family SES not only has a direct impact on learning engagement, but also could have an indirect impact on it through parental autonomous support. Specifically, the higher the family SES, the stronger the parental autonomy support and the stronger the promotion effect on students’ learning engagement.

The study found that family SES had a direct positive effect on learning engagement without considering parental autonomy support. After incorporating parental autonomy support into the structural equation model, it was found that parental autonomy support has a complete mediating effect between family SES and learning engagement. The level of family SES will affect the strength of parental autonomy support. Compared with the parents with high family SES, the parents with low family SES are at a disadvantage in terms of economic conditions and will face more life pressure, so they will have less support for children’s education investment (Conger and Donnellan, 2007). Meanwhile, due to the low educational background of parents, they also lack knowledge on how to give appropriate educational support to their children, which was not conducive to their children’s learning engagement. The research of Davis-Kean (2005) confirmed that parents with high family SES have higher educational level and better economic conditions, and higher education expectations for their children. Therefore, they will pay more attention to their children’s study, and they will be more willing to invest more spiritual and material support in their education. So as to help them devote more time and energy to their studies to the greatest extent. Accordingly, parental autonomy support has a positive impact on students’ learning engagement. According to the SDT proposed by Deci and Ryan (1987), the supportive external environment could meet the basic psychological needs of students. As parents of important others, parental autonomy support could stimulate students’ learning motivation and promote their learning engagement. Therefore, parents should provide supportive learning environment for their children as much as possible to reduce the level of learning engagement caused by different family SES.

### 4.3. Effect of psychological capital on parental autonomy support and learning engagement

In the process of data processing, we found that the interaction term between psychological capital and family SES could not significantly predict learning engagement and parental autonomy support. However, when the interaction between psychological capital and parental autonomy support was tested, it was found that it had a significant positive predictive effect on learning engagement. The moderating effect confirms hypothesis 3 that psychological capital was the moderating variable in the influence of family SES on learning engagement. This study found that psychological capital, as a moderating factor, plays a positive role in promoting the relationship between family SES and learning engagement. When parental autonomy support mediates the relationship between family SES and learning engagement, psychological capital could moderate the second half of the path.

Specifically, the second half of the mediating effect of “family SES—parental autonomy support—learning engagement” was moderated by psychological capital. Compared with students with low psychological capital, students with high psychological capital have more positive effects on learning engagement from their parental autonomy support. This moderation model verifies the promotion hypothesis of the “protective factor—protective factor” model (Zhou and Long, 2004). As an important individual protection factor, psychological capital will promote the impact of another individual protection factor (parental autonomous support) on learning engagement. Psychological capital is an internal positive resource for students, which has the effect of supplementing energy and stimulating motivation. According to COR (Schaufeli and Bakker, 2004), the psychological capital that students have could timely supplement energy and provide resources when consuming energy in the learning process. When students own a high level of psychological capital, sufficient energy is more conducive to supporting them to spend more time and energy in the learning process to continue to invest in the learning field to achieve learning goals.

## 5. Implication

Focus on the learning of low family SES students. The school should give full play to the educational role of the “scholarship loan” policy, provide targeted assistance to low family SES students, alleviate their family’s financial difficulties, and promote them to study harder. Teachers can improve students’ classroom participation, enhance their academic self-efficacy and promote their active learning by organizing rich curriculum practice activities or adopting diversified teaching methods. Parents should establish the concept of self-reliance and self-improvement, constantly create family wealth and wisdom through their hard-working hands, improve their social status, and accumulate economic and social capital for their families.

Create an autonomous support family environment. The influence of family and parents on children is very important, family resources provide conditions for learning, and parental autonomy support affects students’ learning, life and growth. College students, as adults, are individuals with dominant position, independent personality and thoughts. The environment of autonomy support (such as material, emotion and action) provided by parents can make college students feel care, respect and autonomy. It helps them invest more time and energy in learning so as to achieve better academic performance. Therefore, for parents, even if they cannot provide children with adequate financial support, but to give their children understanding, care and support, strengthen communication with their children, enhance parent–child intimacy, and also to help them build confidence in the learning process, students will have better academic performance.

Enhance the psychological capital level of college students. Schools should offer courses related to psychological capital, actively cultivate students’ positive psychological quality, and help students define their objective of the struggle, build up their confidence, and strive to achieve their goals. Through thematic melodrama, group psychological counseling and other positive attribution training for students, increase the positive experience and feeling, and improve the level of students’ psychological capital. Give play to the demonstration role of students with high psychological capital to students with low psychological capital, guide students with low psychological capital to enhance their understanding of the purpose and significance of learning, and improve their level of learning engagement.

## 6. Limitations and future research

This section acknowledges several limitations of this study and points out the direction of future research. First of all, this study only selected college students majoring in preschool education from three schools in Guangdong and Jiangxi provinces of China as the participants, so the representativeness of the study was not strong and the generalization of the research conclusions was insufficient. Subsequent research should select more subjects from other places. Secondly, this study adopts cross-sectional research, which could not fully reveal the causal relationship and stability between variables. Follow up research may combine tracking and intervention research to more deeply reveal the causal relationship between variables. Finally, future studies may focus on how to promote the learning engagement of college students majoring in preschool education of different family SES through family education or counseling intervention, so as to promote their continuous improvement of academic achievement.

## 7. Conclusion

- Family SES could significantly positively predict the learning engagement of college students majoring in preschool education.
- Parental autonomy support plays a completely mediating role between family SES and learning engagement of college students majoring in preschool education.
- The relationship between parental autonomy support and learning engagement of college students majoring in preschool education (the second half of the mediating effect) was moderated by psychological capital, which could promote the positive impact of parental autonomy support on learning engagement.

## Data availability statement

The original contributions presented in the study are included in the article/supplementary material, further inquiries can be directed to the corresponding author.

## Ethics statement

All procedures followed were in accordance with the ethical standards of the responsible Committee on Human Experimentation [Guangzhou University, Guangdong Province, China] and with the Helsinki Declaration of 1975, as revised in 2000. Written informed consent to participate in this study was provided by the participants.

## Author contributions

YQ was mainly responsible for framing the study, proposing the hypothesis, distributing/restoring the questionnaire, collating the questionnaire data, performing statistical analysis and interpretation of the data, and drafting and revising the manuscript. PY was primarily responsible for directing the research, determining the overall framework, and revising and improving the article several times. All authors contributed to the article and approved the submitted version.

## Funding

This work was supported by the Innovation Research Program for Postgraduates of Guangzhou University of China [2021GDJC-D04].

## Conflict of interest

The authors declare that the research was conducted in the absence of any commercial or financial relationships that could be construed as a potential conflict of interest.

## Publisher’s note

All claims expressed in this article are solely those of the authors and do not necessarily represent those of their affiliated organizations, or those of the publisher, the editors and the reviewers. Any product that may be evaluated in this article, or claim that may be made by its manufacturer, is not guaranteed or endorsed by the publisher.
